# The Effects of Live Streamer’s Facial Attractiveness and Product Type on Consumer Purchase Intention: An Exploratory Study with Eye Tracking Technology

**DOI:** 10.3390/bs14050375

**Published:** 2024-04-29

**Authors:** Rui Shi, Minghao Wang, Tongjia Qiao, Junchen Shang

**Affiliations:** 1 School of Economics and Management, Yanshan University, Qinhuangdao 066004, China; shirui@ysu.edu.cn (R.S.); 19831364727@163.com (M.W.); xiaopeng@stumail.ysu.edu.cn (T.Q.); 2 Department of Medical Humanities, School of Humanities, Southeast University, Nanjing 211189, China; 3 College of Psychology, Liaoning Normal University, Dalian 116029, China

**Keywords:** live-streaming e-commerce, facial attractiveness, product type, purchase intention, visual attention

## Abstract

As a booming branch of online retailing, live-streaming e-commerce can present abundant information dimensions and diverse forms of expression. Live-streaming e-commerce has enabled online retailers to interact with customers face-to-face, resulting in widespread instances of emotional and impulse buying behavior. Prior research in live-streaming e-commerce has suggested that live streamers’ characteristics, especially the live streamer’s face, can affect customers’ purchase intentions. The present research used questionnaire surveys and an eye tracking experiment to investigate the impact of live streamer’s facial attractiveness on consumer purchase intention for search-based and experience-based products. The questionnaire survey analyzed 309 valid questionnaires and revealed that attractive faces are the key influencing factor driving consumers’ impulse purchase intentions. Moreover, consumers’ emotional experience plays a partial mediating role in the process of live streamers’ faces influencing purchase intention. The eye tracking experiment further explored the mechanism of a live streamer’s facial attractiveness on consumers’ purchase intentions of search-based products and experience-based products from the perspective of visual attention by analyzing 64 valid sets of data. The results showed that attractive faces attract more consumers’ attention and, therefore, increase their purchase intention. Furthermore, there is a significant interaction between product type, the live streamer’s facial attractiveness, and consumers’ purchase intentions. In the case of unattractive live streamers, consumers are more likely to buy search-based products than experience-based products, while the purchase intention does not vary between search-based products and experience-based products in the case of attractive live streamers. The present study provides evidence for ‘beauty premium’ in live-streaming e-commerce and sheds light on the design of the match between live streamers and different types of products.

## 1. Introduction

With the rapid development of the economy and the internet, the new marketing model of live-streaming e-commerce is well-known to the public through the deep integration and innovation of e-commerce, both physical and online. Data from the 52nd Statistical Report on the Development of the Chinese Internet shows that, as of June 2023, there were 526 million live-streaming e-commerce users in China. Accounting for 48.8% of the overall Internet users. It shows that there is already a huge user base in the field of live-streaming e-commerce [1]. The total merchandise transaction value of live-streaming e-commerce on the whole network in 2023 will be about 4.9 trillion yuan. It accounts for 31.8% of all e-commerce retail sales [2]. Live-streaming social commerce has become an important channel for online shopping and marketing.

Compared with traditional web e-commerce, live-streaming e-commerce enables consumers to obtain more comprehensive product information and make purchasing decisions [3]. Nowadays, live-streaming e-commerce shopping has become an important channel for many consumers to purchase goods, profoundly affecting people’s lifestyles. Live-streaming e-commerce can improve marketing effectiveness. Cheng et al. (2019) established a causal relationship model to study the relationship between live streaming and product sales volume. It was found that merchants using live streaming saw a 21.8% increase in product sales and a significant increase in sales conversion rate [4]. The existing research on live-streaming e-commerce mainly analyzes its impact on consumer purchase intention or behavior from the perspectives of product information, discount promotions, online interaction, etc. Although research on consumer behavior intentions based on the live streamer’s characteristics is more mature, there are shortcomings in the research perspective from the perspective of the live streamer’s externalities, especially facial attractiveness. Product type is a key factor, which has a variety of ways of differentiation in live-streaming contexts. However, the impact of the match between product types and the live streamer’s face in the context of live-streaming social commerce has been somewhat overlooked.

As the most important intermediary between live shopping systems and users, live streamers showcase products in a scenario-based manner through two-way interaction, making “grass-planting” a research focus [5]. The face of the live streamer is the part with high consumer attention. Face is important social information that can be quickly perceived by individuals and affect cognitive processes such as attention [6]. Some studies have found the phenomenon of “profiling” in live-streaming e-commerce scenarios. A spokesperson with an attractive face will produce a “halo effect” and stimulate consumers’ purchase desire [7]. However, the mechanism by which facial attractiveness affects purchase intention is still unclear. The type of product is also an important factor that affects consumer purchasing intentions during live streaming. Different products have their inherent characteristics, which are implicitly stimulating to consumers. Consumers react differently to different types of products, which in turn affects their purchasing behavior [8]. So, does the facial attractiveness of the live streamers’ faces have the same impact on the purchase intention of different types of products? There is currently no definitive conclusion on this issue from existing research. Therefore, this study proposes to use structural equation modeling and eye-tracking experiments to collect objective data from participants for collation and analysis. The aim is to explore the mechanisms by which the facial attractiveness of live streamers influences consumers’ purchase intentions. It also examines the differences in the allocation of consumers attention to different types of products and different live streamer’s facial purchasing processes from a visual attention perspective and provides new solutions for the cost reduction and efficiency of live-streaming e-commerce in the new situation.

## 2. Theoretical Foundations and Hypothesis Development

### 2.1. SOR Theory

Psychologist Woodworth (1926) believed that individuals have two parts: consciousness and behavior. Consciousness plays a mediating role between external stimuli and behavior, thus proposing the SOR model [9]. Mehrabian et al. (1974) improved the SOR model by combining environmental psychology and pointing out that the stimulating effect of the environment affects people’s internal states, prompting individuals to respond [10]. This model is mainly used to explore the effects of stimuli on individual states and conscious responses, as well as the prediction of emotional responses on individual behavior. The SOR model, as a behavioral model, has an expanding range of applications. Donovan (1982) first applied the SOR model to the marketing process and found that store atmosphere, an external stimulus, can change customer psychological perception and lead to purposeful purchasing behavior [11]. At present, the consumption scenario has shifted from offline entities to online e-commerce, and this model can still play a role in providing theoretical support for studying the generation path and influencing factors of user online purchase intention. The SOR model not only incorporates consumer behavior and intentions but also reflects the process of its generation. This includes both external stimuli generated by the environment and internal responses within the individual. This one provides ideas for the study of the scholars. So many scholars use the SOR model for causal analysis in the field of live-streaming e-commerce research. Jakubowska et al. (2023) combined the SOR model to study the impact of consumer characteristics and product knowledge on consumer behavior when purchasing sustainable products [12]. Individual reactions as a necessary condition for behavior have received high attention. Emotional experiences closely related to consumers have become the focus of research. Based on this, this study uses the SOR model to introduce the live streamer’s facial attractiveness, product type as a stimulus (S), consumer emotional experience as an organism (O), and consumer purchase intention (R). Constructing a theoretical framework to explore the intrinsic logic behind the facial attractiveness of live streamers and product types stimulates consumer experience, thereby generating purchase intention.

### 2.2. Facial Attractiveness and Purchase Intention

Face is one of the most frequently encountered visual stimuli in social activities, which can convey rich information and have a significant impact on individual behavior. Facial attractiveness is a key trait of the face and is considered a beneficial social asset [13]. Meanwhile, facial attractiveness is considered important social information. People can improve their decision-making efficiency by processing facial attractiveness to obtain relevant information [14]. The “beauty is good” effect has deeply penetrated people’s hearts. People prefer attractive faces. Different attractive faces have varying degrees of impact on the emotions, attitudes, and behaviors of observers, among which people with attractive faces are generally considered to have good traits [15]. Attractive faces have a reward value, which enhances the activity of reward areas in the brain [16]. Ahmed Shaker et al. (2023) found a positive correlation between facial attractiveness and total compensation, providing strong evidence for the beauty premium [17]. In terms of marketing, Khalid et al. (2019) found that the attractive faces of spokespersons have a positive impact on consumer purchase intention [18]. Moreover, different faces can affect people’s attitudes towards advertisements, their perceptions of brands, and their purchasing intentions, and the impact of faces interacts with the type fof product [19]. And service personnel with attractiveness to consumers will exhibit more customer citizenship behavior, such as willingness to cooperate with service personnel and actively make purchases [20]. Language publishers with beautiful faces in sales scenarios provide consumers with an immersive aesthetic experience, which drives their impulse buying intention [21]. As such, we hypothesize the following:

**H1.**  *In the questionnaire study, live streamers with attractive faces positively influenced consumer purchase intention.*

### 2.3. The Mediating Role of Emotional Experience

The generation of consumer purchase intentions is a complex process. Due to limitations in processing resources, emotions become a key factor affecting decision-making in a short period of time [22]. The theory of emotional evaluation holds that emotional experience is not only an external expression of an individual’s state but also an internal motivation for generating behavior. Individuals who perceive external stimuli will involuntarily subjectively evaluate the attributes of the stimuli and generate emotions related to the stimuli [23], inducing them to choose appropriate behavioral responses [24]. Emotional experience plays a role in explaining individual behavior, enriching research on the causal relationship between emotions and behavior, and has been applied in the field of marketing [25]. Faces have a close connection to individual emotional experiences. Leder et al. (2016) found that attractive faces attracted more attention from individuals, so that they had a more positive emotional experience [26]. Live streamer’s facial expressions have a significant impact on consumers emotional experiences. This emotional arousal is not only at the level of the live streamer’s faces, but consumers will also feel pleasure and reduce their resistance to the shopping environment even when encountering passersby with attractive faces [27]. In marketing practice, Li et al. (2022) found that pleasant emotions have a positive effect on impulsive purchasing behavior among consumers [28]. Many companies are also focusing more on the emotional experience of the consumer. The design of emotionally appealing advertisements can evoke an emotional experience in consumers, which in turn can lead to positive purchasing decisions [29]. Research in the field of cognitive science has also confirmed this. Hahn A.C. et al. (2014) pointed out that when people face attractive faces, they activate the reward system in the brain, generating more positive emotional experience, which can lead to positive evaluation behavior [30]. On the contrary, individuals may become alert when facing negative emotional experiences, which is not conducive to generating proactive behavior. As such, we hypothesize the following:

**H2.**  *Emotional experience plays a mediating role in the relationship between live streamer’s facial attractiveness and consumer purchase intention. And the three variables are positively correlated with each other.*

### 2.4. The Effect of Product Types

The factors involved in the matching of recommenders and products include attractiveness, professionalism, and product type, with attraction and product type being the most widely studied factors. The match-up hypothesis in the field of marketing points out that when the image of the product recommender is consistent with the product, consumers generate a positive review of it [31]. This phenomenon is caused by the difference in stereotype association caused by attractive or unattractive faces [32]. Therefore, the matching between facial attractiveness and products plays a very important role in influencing consumers’ attitudes and purchase intentions. According to Nelson’s (1970) classification, experience-based products are more dependent on subjective attributes, that is, personal taste, while search-based products focus on product quality and objective attributes [33]. For experience-based products, consumers are more likely to perceive the various characteristics that the live streamers present in the process of transmission of information, such as interaction, facial appeal, etc., so that the perception of the live streamers is also more susceptible to these characteristics [34]. Wang et al. (2021) confirmed that live streamers with attractive faces make consumers more focused and immersed in the purchasing process, promoting purchasing behavior. Consumers tend to ignore product attributes when making purchase decisions [35]. Live streamers with unattractive faces shift consumers’ focus to products during the shopping process. Among them, search-based products have objective standards as a reference, and consumers will judge product quality by collecting relevant information and making purchasing decisions based on this [36]. At this point, consumers will be more rational, and the influence of the live streamer’s facial attractiveness will be relatively weakened. While experience-based products have higher uniqueness and lack clear and uniform quality evaluation standards, consumers may have different preferences due to personal circumstances [37]. At this time, consumers’ purchase intention is more dependent on the live streamer’s introduction, and the attractiveness of the live streamer invariably improves the shopping experience and induces a stronger mental simulation. In other words, remembering the past situation of using the product promotes the formation of purchase intention, and the opposite is true for unattractive faces with live streamers. Zhang et al. (2020) considered the adjustable role of product types in live broadcasting, and the impact on perceptual uncertainty is more pronounced than the impact of searching for a product when experiencing a product using a live-streaming strategy [38]. Therefore, this study explores the effect of product types on the impact of a live streamer’s facial attractiveness on consumer purchase intention. As such, we hypothesizethe following:

**H3.**  *In the experimental study, live streamers with attractive faces conditions induced higher consumer purchase intention compared to live streamers with unattractive faces.*

**H4.**  *The effect of facial attractiveness on purchase intention is weaker for search-based products than for experience-based products.*

### 2.5. Eye Tracking Technology

Eye tracking technology is a non-invasive and innovative cognitive neuroscience technology that can capture and record the eye movement processes of consumers browsing product interfaces and form objective data. It has become an effective method for analyzing consumer information processing and revealing consumer behavior. Cognitive psychology research has found that visual attention can reflect the cognitive processing of the brain [39], and cognitive processing theory explains that an individual’s cognitive processing can affect their response to information [40]. Consumers who invest more visual attention can generate more positive product preferences, which in turn affect consumption decisions [41]. Therefore, consumer purchase intention can be studied from the perspective of visual attention. Total fixation durations refer to the browsing time of participants in each area of interest from the beginning of the presentation of an interface to the switch to the next interface, which reflects the depth of information processing and the visual attention of the participants during the browsing of information [42]. In this study, the total fixation durations in the area of interest were selected as an eye movement indicator to measure the cognitive processing of consumers and to analyze the allocation of consumers’ attention to different live streamer’s faces and product types in live e-commerce streaming rooms.

As an active factor in live-streaming e-commerce, the face of the live streamer plays a crucial role. Numerous studies on facial traits have found that facial attractiveness can affect the allocation of attention resources and attract individual attention. Different facial attractiveness can produce different visual attention effects. Liu et al. (2012) found through experimental testing that tracking attractive faces is more accurate than tracking unattractive faces when individuals simultaneously track multiple faces. This result supports individuals generating more attention resources for attractive faces [43]. In the live-streaming e-commerce shopping process, consumers facing live streamers with attractive faces are easily attracted by faces and allocate more cognitive resources. This leads to higher emotional and attentional engagement. Research has found that individuals who notice attractive faces are less likely to experience attentional shifts. Even if there is a transfer, there will be a path return and a re-browsing of the face hole, which greatly improves the fixation duration [12]. When consumers face a live streamer with an unattractive face, they are attracted to it to a lesser extent than an attractive-faced live streamer. At this point, visual attention is distributed to areas other than the face. When a consumer purchases a search-based product, the attributes of the search-based product dictate that the consumer can access relevant information through text or objective data. In turn, the consumer chooses the next behavior, which does not require excessive cognitive effort. At the same time, the product information is focused on the product itself and influenced weakly by the attractiveness of the live streamer’s face. Compared with search-based products, experience-based products have stronger uniqueness. Consumers need to spend more effort processing the information conveyed by the live streamer when introducing the product. Meanwhile, consumers will match the product with themselves to determine whether it meets their expectations. In the online shopping environment, it is difficult for consumers to evaluate the perceptual, tactile, and other attribute information of experiential products through product introductions and other means [44]. Consumers need to go deeper into the live streaming process to experience and analyze the product. This process increases the allocation of consumers’ attention to browse the product and the streamer’s face while watching the live streaming. As such, we hypothesize the following:

**H5.**  *In the experimental study, live streamers with attractive faces conditions induced higher consumers’ total fixation durations compared to live streamers with unattractive faces.*

**H6.**  *The effect of facial attractiveness on total fixation durations is weaker for search-based products than for experience-based products.*

**Figure 1:**
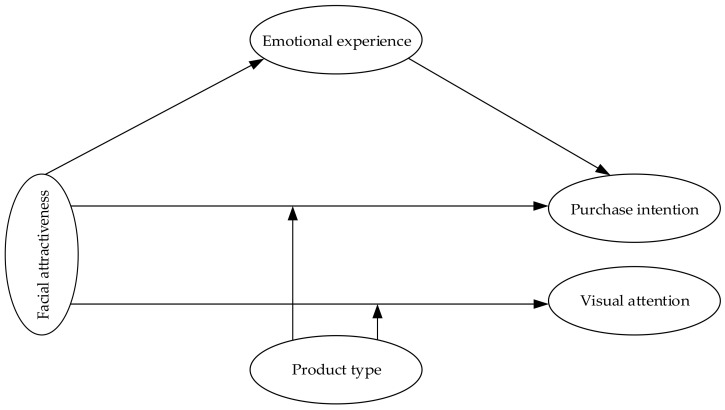
Proposed study model.

Based on the above theories and assumptions, propose the theoretical model of this article, as shown in Figure 1.

## 3. Research Methods and Design

### 3.1. Study 1: Questionnaire Survey

This survey is a preliminary study for the design of the eye tracking experiment. In order to explore the factors influencing consumers’ purchase intentions, the influence of live streamer’s facial attractiveness on consumer purchase intention was analyzed using structural equation modeling.

#### 3.1.1. Survey Instrument

In order to effectively validate the influence path of user purchase intention in live-streaming e-commerce, we designed and modified the questions from previous studies. The questionnaire includes three latent variables: facial attractiveness, emotional experience, and purchase intention. Among them, facial attractiveness referred to the questionnaire scale on personal physical attractiveness in McCroskey’s study and the attractiveness questionnaire scale on advertising spokespersons in Ohanian’s study [45,46]. The emotional experience was referenced in Kim’s questionnaire on consumers’ emotional responses to stimuli in an online shopping environment [47]. The purchase intention was referenced in Li’s questionnaire on consumers’ purchase intention in a live-streaming e-commerce situation [48]. Adapted with the context of live-streaming e-commerce and references. Finally, a final questionnaire was developed.

Each variable is designed with 3–4 questions, totaling 10 items. Detailed items are shown in Table 1. A seven-level Likert scale is a widely used research tool in sociology and management. The scale usually contains seven rating options, each representing a different intensity of attitude. It is a common tool used to measure a participant’s attitude towards a particular issue. The variables in this study were all measured using a seven-level Likert scale method, using integers between 1 and 7 to measure the participants’ attitudes towards the questions. The rating of 1 is strongly disagreeing, and 7 is strongly agreeing. Participants choose based on their actual situation. To ensure the accuracy of the data, a pre-survey was conducted before the official release of the questionnaire. By analyzing the results of the pre-survey data, it was found that this questionnaire can effectively collect the necessary data for the study, ensuring the rationality and authenticity of the research.

#### 3.1.2. Sample and Data Collection

The questionnaire was released online and distributed through WeChat and Credamo sample pools. Participants signed an informed consent form and received a monetary reward for their time. In addition, following the principle of the online survey, we carefully checked the IDs to ensure that each participant could only answer the questionnaire once. At last, a total of 331 questionnaires were collected, of which 309 were valid, with an effective response rate of 93.4%. From the descriptive statistical results, the proportion of males and females in the survey subjects was 46.6% and 53.4%, respectively, indicating a relatively balanced gender ratio. In terms of age, the majority are between 21–30 years old and 31–40 years old, accounting for 58.8% and 33.3%, respectively. From the perspective of educational background, the majority of undergraduate, master’s, and above students are 63.1% and 28.8%, respectively. By analyzing the overall distribution of the survey sample, it is generally consistent with the characteristics of current consumer users on live-streaming e-commerce platforms in China.

#### 3.1.3. Analysis and Discussion of Data Results

##### Reliability and Validity

Due to measurement errors, the reliability and validity of the questionnaire are tested before data analysis. To ensure the scientific validity of the results. Where reliability refers to the degree of reliability of the measured data and validity is the degree of agreement between the observed and latent variables, this study used SPSS 26.0 software to test the reliability and validity, and the data are shown in Table 2. The reliability of the scale was tested using Cronbach’s α coefficient, and the overall questionnaire had a coefficient of 0.884. And the coefficients of each variable are all greater than the standard value of 0.7, indicating that the reliability of the scale is relatively reliable. For the validity test, the KMO value of the output data is 0.883, which indicates that the correlation between variables is strong and suitable for factor analysis. According to the standard value, the factor loading corresponding to all items is greater than 0.5, and the combined reliability (CR) of latent variables is greater than 0.7. The average variance extraction value (AVE) is greater than 0.5, indicating high questionnaire consistency and passing the structural validity test. In addition, discriminant validity was tested by comparing the correlation coefficients between the square root of AVE and the corresponding latent variables. The data indicated that the criteria were met, meaning that the square root of each AVE was greater than the corresponding correlation coefficient, and the discriminant validity passed the test.

##### Path Analysis and Hypothesis Testing

In order to study the impact of a live streamer’s facial attractiveness on consumer perception and purchase intention in live-streaming scenarios, the structural equation model path was first analyzed using AMOS 25.0, and the output was obtained: χ^2^/df = 2.005, RMSEA = 0.057, CFI = 0.977, and GFI = 0.959, all of which meet the standards. This indicates that the research model fits well with the data and is an acceptable model. Secondly, statistical analysis was conducted on the mean, standard deviation, and correlation coefficient of latent variables, as shown in Table 3. The mean and standard deviation of each variable are within an acceptable range. Meanwhile, the correlation coefficients between the variables are all between 0 and 1, indicating a positive correlation between the latent variables in the model. Consumers’ purchase intention is positively and significantly influenced by live streamer’s facial attractiveness. Hypothesis H1 is validated. Moreover, live streamer’s facial attractiveness has a positive effect on consumers’ emotional experiences.

Finally, the Bootstrap method proposed by Hayes (2013) was used for mediating effect analysis [49]. Live streamer’s facial attractiveness is the independent variable; consumer emotional experience is the mediating variable; and consumer purchase intention is the dependent variable. The Bootstrap sample size is set to 5000 and tested using Mode 4 with a 95% confidence interval for significance. The results are shown in Table 4. Of all the mediation paths tested, all paths do not include 0 between the upper and lower limits at a 95% confidence level, indicating significant mediating effects. This direct and mediating effect accounted for 59.81% and 40.19% of the total effect, respectively. Emotional experience partially mediates the impact of a live streamer’s facial attractiveness on purchase intention, and hypotheses H2 are validated.

A systematic analysis was conducted on the mechanism of consumer purchase intention through structural equation modeling. We found that emotional experience has an impact on their purchase intention. On this basis, eye tracking technology is used to explore consumer purchase intention from a visual attention perspective, and the regulatory mechanism of product types in this process is analyzed.

### 3.2. Study 2: Eye-Tracking Experiment

#### 3.2.1. Participants

As college students have enough knowledge and richer experience in online shopping, which is in line with the content of this study, and due to the school environment, college students are a more suitable choice. A total of 69 college students who had shopping experience on e-commerce live-streaming platforms were recruited through a campus market at a university in Qinhuangdao City. Among them, there are 33 males and 36 females, with an age range of 19–23 years old. The proportion of male and female participants in each condition is equivalent. Written informed consent was obtained from each participant before the experiment. All participants were right-handed, with normal vision or corrected vision and normal color vision, and were randomly assigned to two different conditions. Five participants’ data was excluded because their effective sampling rate of eye movement data was less than 70% due to physical shaking and other reasons. The sampling frequency of the participants’ line of sight landing point accounts for less than 70% of the total sampling frequency. A total of 64 valid data samples were ultimately obtained.

#### 3.2.2. Stimuli

The first step is the selection of products. For the selection of certain product types, researchers randomly interviewed 28 college students at a certain university and summarized 10 products that college students often use, including mobile phones, tablets, power banks, textbooks, computers, electric vehicles, backpacks, cotton coats, toilet paper, and shoes. Participants’ judgments on product types were collected on a seven-level questionnaire scale for verification (1 = fully searching, 7 = fully experiential, 4 = neutral). The questionnaire lead-in clearly explains the concept and key differences between experience-based and search-based products. The data was collated and sorted from high to low to clarify the categories of each product type. The two with the highest values were tablets and power banks, and the two with the lowest values were backpacks and cotton jackets. Therefore, tablets and power banks were selected as search-based products, and backpacks and cotton jackets were selected as experience-based products. The classification of this product is basically consistent with existing research [50,51]. The product type classification has a certain degree of scientific validity.

The second step is the selection of live streamer’s faces. The photos of the live streamer’s faces were collected from the internet. All photos are frontal shots, with the figures looking straight ahead. In order to avoid other factors such as clothing and background, we cover the facial hair and ears, retaining only the basic facial features. And recruited 30 college students to test the attractiveness of the live streamer’s face. Photos were randomly presented to the participants. The participants used the Likert seven-level scale to evaluate the attractiveness of the photos (1 = very unattractive, 7 = very attractive). The scores for the attractiveness of each face were averaged to obtain the mean. The results showed significant differences in facial attractiveness (M_attractive_ = 5.133, SD = 0.809; M_unattractive_ = 2.608, SD = 0.614; t = 13.614, *p* < 0.001). Finally, four attractive faces and four unattractive faces were selected as experimental stimuli (with 2 female faces and 2 male faces in each group) to act as live streamers in the live-streaming room.

After determining the product stimuli and the live streamer’s face stimuli, we made sure the experimental stimuli picture finally. The pictures were adopted from the real cargo scenes of the Douyin e-commerce live platform (https://live.douyin.com/ accessed on 14 November 2022). In order to avoid interference with the experimental results from the number of viewers in the live-streaming room and bullet-screen comments, we cleared the number of viewers (upper right corner) and screen comments (lower left corner).

The stimulus materials included clear product information; for the search-based products, we showed the tablets and power banks; except for the product exterior, there was an introduction to specific product models and parameters. For the experience-based products, we showed the backpacks and cotton jackets; except for the product exterior, there was an introduction to the material and manufacturing process of the product. The stimulus materials included clear live streamer face information. We can easily identify the gender and attraction of the live streamer. We ensure that under the same product conditions, only the live streamer’s faces are different, and other positions in the pictures in the live broadcast room are consistent (such as background, shopping cart place, live broadcast room name, etc.). Further, the order of placement was consistent with when consumers purchased products on the live-streaming platform, and the stimulus material was also set to be displayed up and down, in line with the participants’ daily browsing habits of online shopping. At last, we used Photoshop software version 2020 software to edit and process the stimulus picture materials to the same size.

#### 3.2.3. Experimental Design

A 2 × 2 experiment allows us to analyze the results of two independent variables under different conditions. It is used to help the researcher understand the effect of the independent variables and the interaction between them. The study used a 2 (live streamer’s facial attractiveness level: attractive vs. unattractive) × 2 (product type: search-based products vs. experiential-based products) mixed design, with the live streamer’s facial attractiveness level as the subject variable and the product type as the subject variable. Dependent variables are purchase intention based on a subjective questionnaire and total fixation durations in eye tracking data from participants. The area of interest for the experiment was divided into two parts: the live streamer’s faces and merchandise, and the size of the area of interest was ensured to be consistent across different experimental conditions.

**Figure 2:**
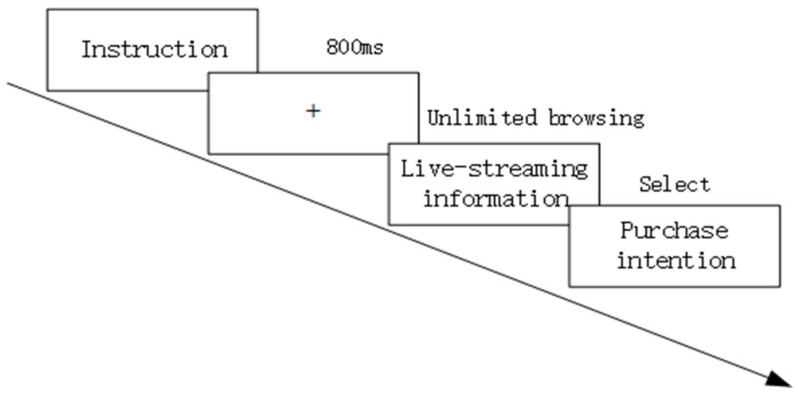
Procedure of Eye Movement Experiment.

The experiment was conducted in the soundproofing laboratory. In this study, we used a SMI-RED 500-Hz device (SensoMotoric Instruments, Teltow, Berlin). The stimuli were presented on a 19-inch computer display with a resolution of 1280 × 1024 pixels. The distance between the screen and the participant’s eyes during the experiment was approximately 60 cm. After explaining the experimental procedure and precautions to the participants, the eye tracking device was adjusted, and a 9-point calibration was performed. Calibration was considered successful when the visual angle of the eye was calibrated within 1°. After passing the calibration, we entered the simulation test and the formal experiment. The monitor presents instructions, imagining the participants purchasing goods for their family in an e-commerce live streaming room and bringing the participants into the experimental context. Each experimental material is presented once and randomly, and the browsing process is not limited by time until a purchase decision can be made. After viewing each image, participants need to choose the Likert seven-level scale (1 = strongly disagreeing, 7 = strongly agreeing) to determine whether they are willing to purchase the product. During the experiment, participants should try not to shake their heads as much as possible to ensure the validity of the data. The experimental process is shown in Figure 2. Each participant was paid appropriately for the experiment at the end of the experiment.

#### 3.2.4. Results

This study further analyzed the purchasing behavior data obtained from participants participating in mixed experiments. A 2 (live streamer’s facial attractiveness: attractive vs. unattractive) × 2 (product type: search-based products vs. experience-based products) repeated measurement analysis of variance was performed on behavioral data and eye tracking data. The results of consumer purchase intention and total fixation durations are as follows:

##### Results of Behavior Data

**Figure 3:**
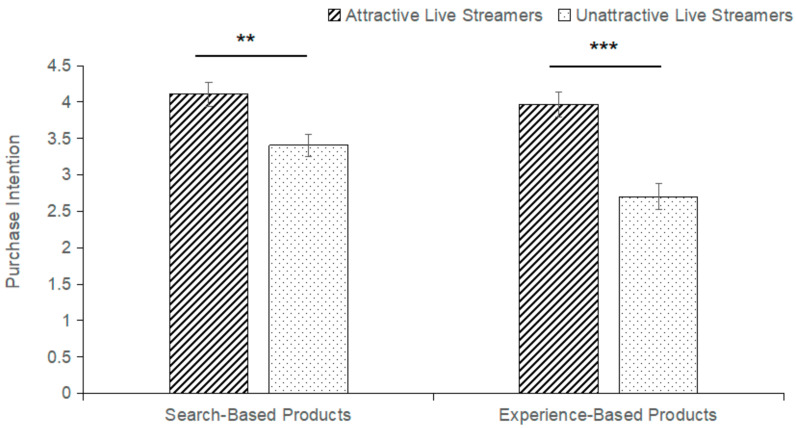
Consumer behavioral data from eye-tracking experiments (*** *p* < 0.001, ** *p* < 0.01).

The results showed a significant main effect of product type (F(1,62) = 10.693, *p* = 0.002, η^2^_P_ = 0.147). Consumers’ purchase intention for search-based products in the live-steaming e-commerce environment was significantly higher than that for experience-based products (M_search-based_ = 3.758, SE_search-based_ = 0.120; M_experience-based_ = 3.336, SE_experience-based_ = 0.145). The main effect of a live streamer’s facial attractiveness on consumer purchase intention is significant (F(1,62) = 25.143, *p* < 0.001, η^2^_P_ = 0.289). And consumers’ purchase intentions were significantly higher for live streamers with attractive faces than for live streamers with unattractive faces (M_attractive_ = 4.039, SE_attractive_ = 0.118; M_unattractive_= 3.055, SE_unattractive_ = 0.124), hypothesis H3 is validated. In addition, it was found that the interaction between a live streamer’s facial attractiveness and product type on consumer purchase intention is significant (F(1,62) = 4.752, *p* = 0.033, η^2^_P_ = 0.071). On this basis, we conducted a simple effect analysis. Consumers purchased both search-based products (M_attractiveness_= 4.109, SE_attractiveness_ = 0.165; M_unattractiveness_ = 3.406, SE_unattractiveness_ = 0.153; *p* = 0.003) and experiential products (M_attractiveness_ = 3.969, SE_attractiveness_ = 0.169; M_unattractiveness_ = 2.703, SE_unattractiveness_ = 0.177; *p* < 0.001). The live streamer’s facial attractiveness has a significant effect on consumer purchase intention. Therefore, hypothesis H4 was not validated. There was no significant difference in purchase intention between search-based and experience-based products when facing live streamers with attractive faces (M_search-based_ = 4.109, SE_search-based_ = 0.165; M_experience-based_ = 3.969, SE_experience-based_ = 0.169; *p* = 0.444). When faced with live streamers with unattractive faces, there is a significant difference in consumers’ purchase intention between search-based and experience-based products (M_search-based_ = 3.406, SE = 0.153; M_experience-based_ = 2.703, SE_experience-based_ = 0.177, *p* < 0.001). And consumer purchase intention for search-based products is significantly higher than that for experience-based products. It is shown in Figure 3.

##### Results of Eye Movement Data

For the purpose of verifying whether a live streamer’s facial attractiveness has an effect on total fixation durations in different areas of interest, the total fixation durations of eye movement indicators were processed in this study. There were two areas of interest: the live streamer’s face area of interest (AOI 1) and the product area of interest (AOI 2).

The main effect of product type on face AOI (F(1,62) = 13.992, *p* < 0.001, η^2^_P_ = 0.184) and product AOI (F(1,62) = 21.731, *p* < 0.001, η^2^_P_ = 0.260) total fixation durations was significant. Consumers spent significantly more time looking at face AOI (M_search-based_ = 1.135, SE_search-based_ = 0.058; M_experience-based_ = 1.012, SE_experience-based_ = 0.057) and product AOI (M_search-based_ = 1.859, SE_search-based_ = 0.064; M_experience-based_ = 1.650, SE_experience-based_ = 0.066) than at experiential products when purchasing search-based products. The main effect of live streamer’s facial attractiveness on face AOI (F(1,62) = 9.907, *p* = 0.003, η^2^_P_ = 0.138) and product AOI (F(1,62) = 12.364, *p* < 0.001, η^2^_P_ = 0.166) total fixation durations was significant. As compared with live streamers with an unattractive face, face AOI (M_attractive_ = 1.236, SE_attractive_ = 0.060; M_unattractive_ = 0.912, SE_unattractive_ = 0.048) and product AOI (M_attractive_ = 1.950, SE_attractive_ = 0.058; M_unattractive_ = 1.558, SE_unattractive_ = 0.065) with attractive live streamers will receive more attention from consumers. Hypothesis H5 is validated. Besides, the results found a significant interaction between the live streamer’s facial attractiveness and product type, so the simple effects of attractive and unattractive live streamer’s face on the attention time of different areas of interest were analyzed separately.

**Figure 4:**
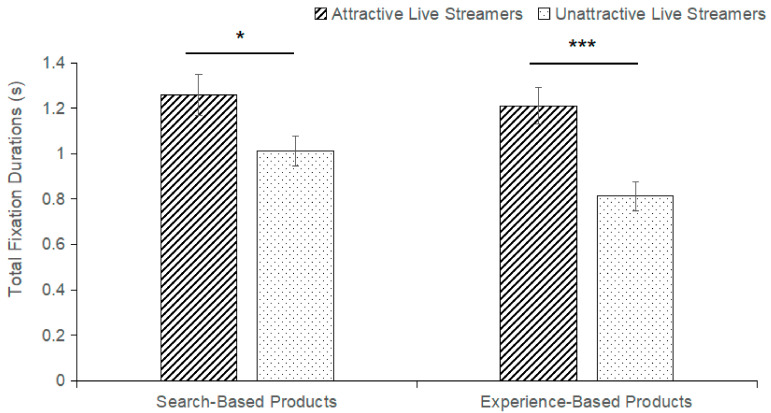
Total Fixation Durations in the Live Streamers’ Face Area of Interest (*** *p* < 0.001, * *p* < 0.05).

The interaction between the live streamer’s facial attractiveness and product type has a significant impact on the total fixation durations of the face AOI (F(1,62) = 5.161, *p* = 0.027, η^2^_P_ = 0.077). The data found that regardless of whether consumers purchased a search-based product (M_attractiveness_ = 1.260, SE_attractiveness_ = 0.091; M_unattractiveness_ = 1.011, SE_unattractiveness_ = 0.067; *p* = 0.031) or an experiential product (M_attractiveness_ = 1.211, SE_attractiveness_ = 0.080; M_unattractiveness_ = 0.813, SE_unattractiveness_ = 0.064; *p* < 0.001), there was a significant effect of live streamer’s facial attractiveness on the amount of time consumers spent gazing at the face AOI. Within the group of attractive faces, different product types have no significant impact on the total fixation durations of the live streamer’s face AOI (M_search-based_ = 1.260, SE_search-based_ = 0.091; M_experience-based_ = 1.211, SE_experience-based_ = 0.080, *p* = 0.303). It means that consumers’ total fixation durations to face AOI are not affected by product type when facing live streamers with attractive faces. Within the group of unattractive faces, product type has a significant impact on the total fixation durations of live streamer’s face AOI (M_search-based_ = 1.011, SE_search-based_ = 0.067; M_experience-based_ = 0.813, SE_experience-based_ = 0.064; *p* < 0.001). It means that when facing live streamers with unattractive faces, the consumers’ total fixation durations for the face AOI are influenced by the product type. The search-based product has a significantly higher influence than the experience-based product in this process. It is shown in Figure 4.

**Figure 5:**
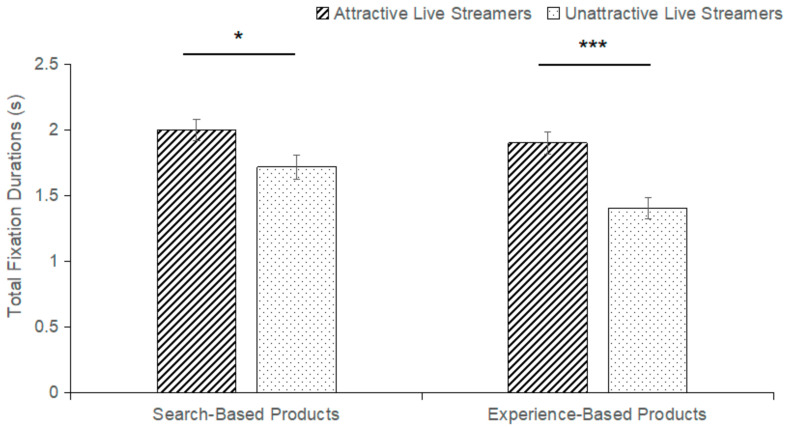
Total Fixation Durations in Product Areas of Interest (*** *p* < 0.001, * *p* < 0.05).

The interaction between live streamer’s facial attractiveness and product type on the total fixation durations of product AOI was significant (F(1,62) = 5.677, *p* = 0.02, η^2^_P_ = 0.084). The data found that regardless of whether consumers purchased a search-based product (M_attractiveness_ = 2.001, SE_attractiveness_ = 0.081; M_unattractiveness_ = 1.716, SE_unattractiveness_ = 0.093, *p* = 0.025) or an experiential product (M_attractiveness_ = 1.899, SE_attractivenes_ = 0.082; M_unattractiveness_ = 1.401, SE_unattractiveness_ = 0.082, *p* < 0.001), there was a significant effect of the live streamer’s facial attractiveness on product AOI. Within the group of attractive faces, the effect of different product types on the total fixation durations of product AOI is not significant (M_search-based_ = 2.001, SE_search-based_ = 0.081; M_experience-based_ = 1.899, SE_experience-based_ = 0.082, *p* = 0.112). It means that when facing live streamers with attractive faces, the consumers’ total fixation time on the product AOI is not affected by the product type. Within the group of unattractive faces, product type has a significant impact on the total fixation durations of product AOI (M_search-based_ = 1.716, SE_search-based_ = 0.093; M_experience-based_ = 1.401, SE_experience-based_ = 0.082, *p* < 0.001). That is, when facing live streamers with unattractive faces, the consumers’ product AOI total fixation durations are influenced by the product type. And search-based products have significantly higher influence than experience-based products in this process. It is shown in Figure 5.

In summary, the effect of a live streamer’s facial attractiveness on consumer attention time in a live e-commerce context is independent of product type; hypothesis H6 is not validated.

## 4. Discussions

### 4.1. The Relationship between Facial Attractiveness, Emotional Experience, and Purchase Intention

The results of structural equation modeling remain consistent with the hypothesis. The questionnaire results indicate that a live streamer’s facial attractiveness in a live-streaming e-commerce context can directly influence consumers’ purchase intentions. This is consistent with previous research on consumer behavior. In addition, it is also possible to influence consumer individual status to generate purchase intention, and consumer emotional experience plays a partial mediating role in this process. The hypothesis of this study is consistent with the actual situation. People show higher levels of pleasant emotional feelings toward individuals with attractive faces [52,53]. Implemented in the live-streaming e-commerce environment, consumers pleasant emotions can rise from the live streamer level to the product and shop level. Live streamers with attractive faces can bring positive emotional experiences to consumers, who are more likely to emotionally empathize with the live streamers. Consumers’ shopping emotions may be aroused, which in turn leads to purchase intention. Furthermore, the pleasant emotional process reduces the perceived risk in the purchase process. It can act directly on consumers’ purchase intentions. This provided support for the validation of the intermediary.

### 4.2. The Effect of Facial Attractiveness and Product Type on Purchase Intention

Based on behavior data from eye-tracking experiments, it was found that the type of products sold by live streamers plays a role in the process of consumers’ purchase intention. At the same time, the facial attractiveness of live streamers significantly increased consumers’ purchase intentions. Specifically, consumers are more willing to purchase search-based products in live-streaming e-commerce contexts. This is related to the attribute of search-based products: that information can be easily and quickly understood. However, experience-based products need to be experienced by consumers before they can develop individual perceptions of them. The virtual shopping environment is not conducive to direct experience, which affects consumers’ next behavior. Consumers are more likely to be attracted to and reduce their judgment of product attributes when facing live streamers with attractive faces. Product type has no significant effect on purchase intention at this point. Consumers’ purchase intention is more likely to be affected by the knock-on effect of the live streamer’s attractive faces, which increases the sense of shopping experience. In front of live streamers with an unattractive face, consumers focus on the product. The purchase process will be more rational. Compared with experience-based products, the characteristics of search-based products are easier to understand by consumers through the introduction of products, and the degree of purchase intention is less affected by the face of the live streamers. At this time, product type has a significant effect on consumer purchase intention. And the purchase intention is higher for search-based products.

### 4.3. The Effect of Facial Attractiveness and Product Type on Consumer Visual Attention

Attention is a scarce resource and a precious asset in the era of the Internet [54]. Live-streaming e-commerce, as an important form of attention economy, involves the production and control of consumer attention [55]. Analyzing consumer visual attention is of great significance for studying live-streaming e-commerce. Therefore, by analyzing eye tracking data, we can determine consumers’ visual attention allocation when purchasing goods in e-commerce live streaming rooms. Research has found that live streamers’s faces and products are the areas that consumers focus on. Live streamers play a leading role in the live-streaming sales process and are an active element in the live-streaming context. Consumers are influenced by a live streamer’s face when allocating cognitive resources.

Based on eye movement data from eye-tracking experiments, it was found that the interaction between a live streamer’s facial attractiveness and product type has a significant impact on the total fixation durations of the face AOI and product AOI. We found that consumers’ total fixation durations to face AOI are not affected by product type when facing live streamers with attractive faces. But within the group of unattractive faces, product type has a significant impact on the total fixation durations of the live streamer’s face AOI. That is consistent with the research results. Consumers have limited cognitive resources to process information and tend to pay less cognitive cost to simplify their decision-making motivation in a shopping environment. Search-based products happen to provide clear parameters for consumers to evaluate, thus increasing attention time [56]. However, experiential products require consumers to incur additional cognitive costs to obtain product information, leading to reduced attention [57]. This result is inconsistent with hypothesis H6, which may be due to the fact that we used a different research methodology (eye tracking technology) than in the past. Attractive faces attract more consumers’ attention, whether search-based products or experience-based products, which increase their purchase intention. Analyzed by live streamers’s facial attractiveness, live streamers with attractive faces attracted more consumer attention and reduced visual attention to the product. Live streamers with an unattractive face caused participants to fixate less on both the product and the face. This comes from the fact that people prefer attractive faces during the shopping process. This makes people less likely to be attracted to individuals with unattractive faces and the products they introduce. This comes from the fact that people prefer attractive faces during the shopping process. This makes people less likely to be attracted to individuals with unattractive faces and the products they introduce. The search-based products provide access to more accurate product quality parameters compared with experiential products [58]. This process requires consumers to devote more visual attention to obtaining product information, thus significantly increasing total fixation durations.

## 5. Conclusions and Suggestions

### 5.1. Conclusions

In this paper, we adopt a questionnaire survey and eye tracking experimental method to study the live streamer’s facial attractiveness in the live-streaming e-commerce scene. The aim is to explore how the facial attractiveness of live streamers affects consumers’ purchase intentions and the role that product type plays in this process. It also explores the cognitive processing process behind it from the perspective of visual attention. In the current rapid development of live-streaming e-commerce, this study provides a useful exploration of revenue enhancement. The main conclusions are as follows:

Firstly, from the point of view of consumers’ purchase intentions, this study confirms that ‘beauty premium’ does exist in the context of live-streaming e-commerce. This may have something to do with the mate selection preferences that people have developed over the course of their long evolutionary history [59]. Consumers are more likely to favor live streamers with a better physical appearance. Highly attractive facial features can bring more advantages. Consumers will develop trust in them, as well as confidence in their expertise and advice [60]. Live streamers facial attractiveness has a significant effect on consumer purchase intention. And the attractiveness of the live streamer’s face can significantly increase consumer purchase intention. At the same time, there is a significant gap between the degree of influence of live streamers with unattractive faces on consumers’ purchase intentions and live streamers with attractive faces.

Secondly, it explored the formation mechanism of purchase intention from the perspective of the user. It was found that live streamers with attractive faces as external stimuli can enhance consumers’ emotional experience in the whole shopping process. At the same time, consumer emotions act as a stimulus, influencing their trust in the business environment and further guiding the consumer’s behavioral intentions [61]. That is, it can influence the formation of purchase intentions.

Finally, through eye tracking experiments, we intuitively discovered that consumers pay more attention to the live streamer’s face and product in the purchase process than in other regions. As an important component in live e-commerce, product type has a certain impact on consumers’ purchase intentions, and consumers preferred to purchase search-based products. There is a difference in the impact of product type on consumer purchase intention in the presence of different live streamers’s faces. There is no significant effect of product type on consumers’ purchase intentions when they face attractive faces. At this time, there was no significant difference in the total fixation duration of consumer attention to the product. When facing live streamers with unattractive faces, product type has a significant effect on consumers’ purchase intentions. The total fixation durations for search-based products were higher than those for experience-based products.

### 5.2. Suggestions and Research Limitations

This study supplements and improves the theoretical mechanism of consumer purchase intention under new marketing methods. On the theory side, this study expands the research on the challenges live streamers face in the field of live-streaming e-commerce. This provides new research ideas for consumer behavior research from the perspective of live streamers. At the same time, product types were introduced, which combined consumer, live streamer, and product research, making consumer research more specific. On the practical side, it proposes feasible guidance for merchants or e-commerce platforms from the perspective of the live streamer’s face and product type adaptation. This will further increase the marketing revenue of the merchants’ live streaming of products.

We did not investigate the moderating effect of product type in the survey, which is a limitation. However, the findings of the eye-tracking experiment suggested paying attention to the match between the live streamers and the product. And merchants should fully assess the live streamer external and product type comprehensively before carrying out live banding. For example, it can be appropriately adapted to sell as a live stream with unattractive faces when selling a search-based product. It focuses more of the consumers’ attention on the product, which gives them more space to experience it. In addition, it is possible to carry out a special live sale, distinguishing between search-type products and experience-based products for sale separately. This provides a more immersive purchasing environment and improves the efficiency of the rational allocation of live streamer resources.

Enhance the customer experience in the marketing process. The formation of consumer purchase intentions is influenced by individual emotional experiences. Therefore, the marketing process should focus on enhancing the customer experience. Through various ways to improve the consumer’s positive emotional experiences. For example, it is necessary to improve the professionalism of the live streamers and provide them with regular marketing training. Actively interact with consumers to improve the overall atmosphere of the live streamers. In addition to setting up a reward mechanism to enhance the consumer’s emotional experience, there is an all-round improvement in the effective collocation of people, fields, and things in the live streaming room to improve marketing revenue.

This study effectively explores consumer purchase intention in a live-streaming e-commerce environment through eye-tracking technology. At the same time, this study had several experimental limitations, as follows: Firstly, most of the participants selected for the eye tracking experiment were college students. Although they have experience purchasing products on live streaming platforms, there are many factors that affect their purchases due to their lack of economic independence. This can easily lead to incomplete data. In the future, the sample size should be expanded to improve the universality of conclusions. In addition, no experimental design or analysis has been conducted on the matching of product types and live streamer gender. Targeted research can be conducted on live streamer gender in the future.

## Figures and Tables

**Table 1:** Specific measurement items.

Latent Variable	Measurement Question	
Facial attractiveness	I would pay more attention to live streamers with attractive faces in the live streaming room.	
	The beautiful face of the live streamers are highly appealing to me when I purchase items in the live streaming room.	
	The attractive face of live streamers in an e-commerce live stream room draws my attention to what he is publishing.	
Emotional experience	Below are four descriptive emotional state words. Please rate each emotional word based on your actual emotional experience during the time you watched the live streaming and purchased the product in the live streaming room.	Happy
		Relaxed
		Excitedly
		Lively
Purchase intention	I am willing to purchase items while watching the live stream.	
	I would like to follow or buy related products recommended by the live streamers.	
	I would suggest people around me to shop with me inside the streaming room.	

**Table 2:** Confirmatory factor analysis results.

	Question Items	Standard Load	Cronbach’ α	CR	AVE
Facial attractiveness	q1	0.75	0.827	0.8265	0.6138
	q2	0.82			
	q3	0.78			
Emotional experience	q4	0.8	0.821	0.8279	0.5474
	q5	0.65			
	q6	0.74			
	q7	0.76			
Purchase intention	q8	0.76	0.824	0.8247	0.6107
	q9	0.78			
	q10	0.8			

**Table 3:** The Mean, Standard Deviation, and Correlation Analysis Results of Variables.

Variable	M	SD	1	2	3
1. Facial attractiveness	5.60	0.85	—		
2. Emotional experience	5.56	0.73	0.57 **	—	
3. Purchase intention	5.56	0.87	0.34 **	0.55 **	—

Note: ** *p* < 0.01, “—” means the coefficient between the same variables is 1.

**Table 4:** Mediation Effect Values and Effect Quantity.

	Intermediary Path	Effect Value	Effect Proportion	Bootstrap 95% CI	
				LLCI	ULCI
Total effect		0.5513		0.4490	0.6540
Direct effect	Facial attractiveness → Purchase intention	0.3297	59.81%	0.2118	0.3850
Intermediary effect	Facial attractiveness → Emotional experience → Purchase intention	0.2216	40.19%	0.0314	0.1319

## Data Availability

The data are available from the authors upon reasonable request.
